# *Aeromonas veronii* Infection in Commercial Freshwater Fish: A Potential Threat to Public Health

**DOI:** 10.3390/ani10040608

**Published:** 2020-04-02

**Authors:** Tong Li, Sayed Haidar Abbas Raza, Bintong Yang, Yufeng Sun, Guiqin Wang, Wuwen Sun, Aidong Qian, Chunfeng Wang, Yuanhuan Kang, Xiaofeng Shan

**Affiliations:** 1 College of Animal Science and Technology, Jilin Provincial Engineering Research Center of Animal Probiotics, Key Laboratory of Animal Production and Product Quality Safety of Ministry of Education, Jilin Agricultural University, Changchun, Jilin 130118, China; LiTong775232812@163.com (T.L.); gracebintong@163.com (B.Y.); sunyufeng971212@163.com (Y.S.); wgqjlau@aliyun.com (G.W.); sunwuwen211@163.com (W.S.); qianaidong0115@163.com (A.Q.); wangchunfeng@jlau.edu.cn (C.W.); 2 College of Animal Science and Technology, Northwest A&F University, Yangling 712100, China; haiderraza110@nwafu.edu.cn; 3 College of Life Science, Changchun Sci-Tech University, Shuangyang District, Changchun 130600, China

**Keywords:** *Aeromonas veronii*, freshwater fish, virulence genes, pathogenicity test

## Abstract

**Simple Summary:**

*Aeromonas veronii* is an important aquatic zoonotic agent. In this study, *A. veronii* was isolated from healthy fish, and the relationship between the pathogenicity and virulence genes of *A. veronii* was investigated by molecular identification. The aim of this study was to ensure the safety of freshwater products by evaluating the infection status in edible freshwater fish.

**Abstract:**

*Aeromonas veronii* is an important pathogen causing freshwater fish sepsis and ulcer syndrome. An increasing number of cases have demonstrated its significance as an aquatic zoonotic agent. The purpose of this study was to ensure the safety of freshwater products by evaluating the infection status of edible freshwater fish. In this experiment, we isolated *A. veronii* from several species of apparently healthy freshwater fish, including *Carassius auratus*, *Cyprinus carpio*, *Ctenopharyngodon idella*, and *Silurus asotus*. *A. veronii* was identified through bacterial staining, culture characteristics, and 16S rDNA gene sequence. In addition, polymerase chain reaction (PCR) was used to investigate the distribution of seven major virulence genes, including aerolysin (*aer*: 88.51%), cytotoxic enterotoxin (*act*: 71.26%), serine proteinase (*ser*: 54.02%), adhesin (*Aha*: 40.23%), phospholipase (*lip*: 45.98%), nuclease (*exu*: 51.72%), and quorum sensing-controlled virulence factor (*LuxS*: 59.77%). In total, 496 strains of *Aeromonas* were isolated, including 87 strains of *A. veronii*. The isolates of *A. veronii* were Gram-negative, rod-shaped bacteria, and the colonies are yellow on Rimler-Shotts (RS) medium and showed greater than 99% homology with *A. veronii* ATCC35624 according to analyses of the 16S rDNA sequence. Nearly 50% of the *A. veronii* isolates carried at least four or more virulence genes, 25% of the isolates carried at least five types of virulence genes, and 59.77% isolates carried the *LuxS* gene, and the isolates carrying more virulence genes were found to be more virulent. These results are of great significance for further improving the food safety assessment of freshwater aquatic products.

## 1. Introduction

*Aeromonas veronii* is a Gram-negative, rod-shaped, and facultative anaerobic bacterium. *A. veronii* is widely distributed in nature, with strong environmental adaptability [1]. In recent years, there have been an increasing numbers of cases of large-scale *A. veronii* outbreaks. It has been reported that *A. veronii* can infect freshwater fish, amphibians, birds, and red meat animals, resulting in serious losses to the aquaculture industry and threatening food safety [2,3,4]. In addition, *A. veronii* can also cause human infections, especially in elderly and children with low immunity, causing sepsis, gastroenteritis, and other diseases [5,6,7,8,9,10,11]. Recent reports suggest that individuals with healthy immune function can be infected [12,13]. In recent years, the development of aquaculturehas been accompanied by an increased incidence of bacterial diseases. The abuse of antibiotics has led to the increased antibiotic resistance of *Aeromonas*, and the presence of antibiotic residues in aquatic products threatens human health [14]. In recent years, reports of infectious diarrhea and food poisoning caused by pathogenic bacteria have increased [15].

Previous research shows that the pathogenicity of *Aeromonas* involves many factors that work together. There has been a variety of proven virulence factors, including outer membrane proteins, motility related factors, toxins, proteases, quorum sensing systems, secretion systems, and iron ion acquisition systems, among others [16,17]. Related research demonstrated that hemolytic and cytotoxic activities and pathogenicity were correlated with the presence of virulence genes in *Aeromonas* strains [4]. Those virulence factors play an important role in the development of disease. In this study, seven important virulence genes (*aer, act, Aha, ser, exu, lip,* and *LuxS*) of the isolates were detected by PCR assay.

*Carassius auratus*, *Cyprinus carpio*, *Ctenopharyngodon idella*, and *Silurus asotus* are some of the most common edible fish in northern China. In 2018, the total output of freshwater fish in China was 25.44 million tons, including 2.77 million tons of *Carassius auratus*, 2.96 million tons of *Cyprinus carpio*, 5.50 million tons of *Ctenopharyngodon idella*, and 0.37 million tons of *Silurus asotus* (China Fishery Statistical Yearbook, 2019). The production of these four types of fish comprises nearly half of all freshwater fish production. Therefore, ensuring the safety of these aquatic products is vital to protecting consumer health. Hence, the investigation of *A. veronii* infection is significant from a public health perspective. Healthy freshwater fish of the four common species, available in Changchun City, were used as research materials. *A. veronii* were isolated from these fish followed by an investigation of virulence genes in the bacterial isolates. The results of this study will be of great significance for further improving the food safety assessment of freshwater aquatic products.

## 2. Materials and Methods 

### 2.1. Sample Information

In the spring (April to May) and summer (June to August) of 2014, we sampled 203 apparently healthy freshwater fish from five large supermarkets and five aquaculture markets in the city of Changchun. Details regarding the sampling of fish, including *Carassius auratus* (length 20 ± 2 cm, weight 300 ± 100 g), *Cyprinus carpio* (length 40 ± 5 cm, weight 1500 ± 500 g), *Ctenopharyngodon idella* (length 60 ± 5 cm, weight 1500 ± 500 g), and *Silurus asotus* (length 50 ± 5 cm, weight 2000 ± 500 g) are presented in Table 1 and Table 2. All fish were reared in flow-through aquariums (200 L) at an average temperature of 26 ± 1.0 °C, with a natural photoperiod. Fish were fed with a commercial diet corresponding to an amount of 2% of their body weight twice per day. During the entire experimental period, 30% of the water in each tank was exchanged daily, and the pH was kept at 7.8 ± 0.5, dissolved oxygen at 5.6 ± 0.45 mg/L, nitrate at 0.015 ± 0.003 mg/L, and ammonia at 0.12 ± 0.01 mg/L. 

### 2.2. Ethics Statement

Healthy freshwater fish were purchased from large supermarkets and aquaculture markets in Changchun City in China. Additionally, the mice used for pathogenicity experiments were purchased from the experimental animal center of Jilin University in China (JLAU08201409). All animal protocols were reviewed and approved by the animal administration and ethics committee of Jilin Agricultural University. The study was performed in strict accordance with the recommendations set forth in the animal ethics procedures and guidelines of the People’s Republic of China.

### 2.3. Separation, Purification, and Physiological and Biochemical Test

We performed anesthesia and surface disinfection on the selected fish. Fish intestinal contents were scraped by aseptic cotton buds, inoculated in alkaline peptone water under sterile conditions, and cultured at 28 °C for 6 h. The enriched bacterial cultures were streaked onto RS identification medium (Beijing Bridge Technology Co., Ltd., Beijing, China) and cultured at 28 °C for 18 h. Next, 3–5 characteristic colonies were randomly picked for inoculation in nutrient broth (Beijing Landbridge Technology Co., Ltd., Beijing, China) and amplified for 18 h. Then, we streaked them out again on RS culture medium, and repeated the process. In addition, the colonies were Gram-stained and microscopically examined. The cultures were stored at 4 °C.

The purified bacteria were inoculated in a microbiological reaction tube (Hangzhou Microbe Reagent Co., Ltd., Hangzhou, China) according to the manufacturer’s specifications. We conducted a preliminary identification of strains, and the test results were referenced using the System Identification Manual of Common Bacteria and Bergey’s Manual of Systematic Bacteriology [18,19]. 

### 2.4. Molecular Sequencing

The identified strains were initially inoculated in nutrient broth and cultured for amplification. The extraction of bacterial genomic DNA was performed according to the manufacturer’s instructions (Beijing Solarbio Cable Technology Co., Ltd., Beijing, China), followed by preservation at −20 °C. Then, we searched the 16S rDNA gene sequences of different *Aeromonas* species in GenBank, using specific primers verified by BLASTn (Table 3) [20]. The PCR system (25 μL) included 0.5 μL template (bacterial genomic DNA), 2.5 μL 10 × PCR Buffer, 0.5 μL 5 U/L TaqDNA polymerase, 2 μL 2mmol/L dNTPs, 10 μM primers (0.5 μL each), and ddH_2_O. The ATCC35624 genomic DNA (template) of the standard *A*. *veronii* strain served as the positive control, and ATCC7966 genome DNA (template) of *A. hydrophila* represented the negative control. Reaction conditions were as follows: 94 °C for 5 min; 30 cycles of 94 °C for 30 s, 56 °C for 30 s, and 72 °C for 45 s; 72 °C for 10 min; and a final temperature of 4 °C. The bacterial strain was identified using 1% agarose gel electrophoresis. The positive strains were identified by the presence of a single positive band of 886 bp, and amplicons were sent to TaKaRa (Dalian, China) for sequencing after purification by gel extraction. The sequencing results were identified using BLAST searching. The accession number of the 16s rDNA gene for reference strain was *A. veronii* ATCC35624 (X60414.2).

### 2.5. Virulence Gene Detection

Based on published studies and GenBank sequences, seven pairs of specific primers were designed (Table 3) [21]. To determine the genomic DNA template in the PCR reaction system, we investigated seven virulence genes in 87 strains of *A. veronii*, with ATCC35624 genomic DNA as the positive control and standard strains serving as the template, and performed PCR followed by agarose gel electrophoresis. The PCR products of different virulence genes in several bacterial isolates were randomly selected. These PCR products were recovered using the DNA gel extraction kit according to the instructions provided by Axygen Biotechnology Co., Ltd. (Hangzhou, China). Purified products were sent to Takara for sequencing. The sequencing results were analyzed using BLAST to verify the accuracy of the PCR results.

### 2.6. Pathogenicity Test on Mice

The pathogenic experiments on mice were carried out by randomly selecting strains of *A. veronii* carrying different virulence genes. Bacteria were isolated from the dead mice and identified to confirm that the pathogenic strain was *A. veronii*, and the LD50 was calculated using the Reed and Muench Method [22]. 

## 3. Results

### 3.1. Bacterial Separation, Purification, and Physicochemical Identification

Using RS medium for growth, we isolated 496 strains that were round, smooth, and moist, and colored yellow or yellow-green, suggesting that they were *Aeromonas* strains. Identification based on published methods showed that 91 of these putative isolated *Aeromonas* strains correspond to strains of *A*. *veronii* [23].

### 3.2. Molecular Identification

**Figure 1:**
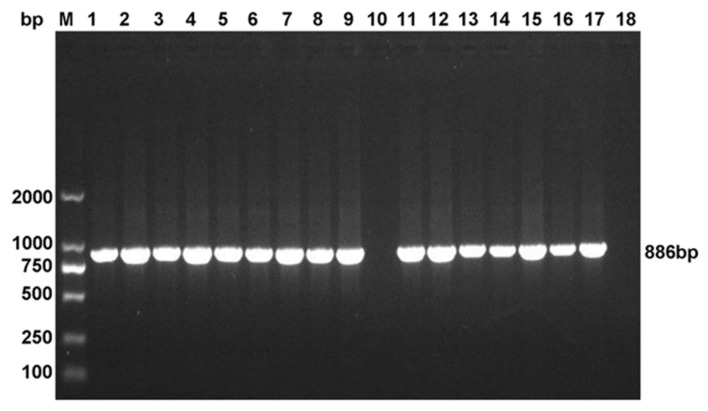
Detection of 16S rDNA gene in isolates by PCR. M: DNA molecular weight standard. 1–16: PCR of 16S rDNA gene in bacteria isolates; 17: positive control; 18: negative control.

We confirmed the identity of any isolates initially suspected to be *A. veronii* strains through the PCR of 16S rDNA using specifically designed primers, and the strains were determined as potential positive strains if the electrophoretic band appeared at about 886 bp (Figure 1). The positive PCR products were confirmed following gel extraction, purification, sequencing, and sequence comparison with BLASTn. The isolates of *A. veronii* showed greater than 99% homology with *A. veronii* ATCC35624(X60414.2) by analysis of the 16S rDNA sequence. Combined with the physiological and biochemical test results (Table S1), we finally identified 87 strains of *A. veronii*, accounting for 17.54% of the isolated strains.

The separation rate (number of *A. veronii* isolates/total isolates) of *Carassius auratus* was the highest, at 23.44%. The separation rates of *Cyprinus carpio* and *Ctenopharyngodon idella* were 21.49% and 18.85%, respectively. The separation rate of *Silurus asotus* was the lowest, at 6.40%. The differences in sampling times and locations, and the conditions of different fish carrying the identified 87 strains are presented in Table 4. 

### 3.3. Detection of Virulence Genes in A. veronii Isolates 

In addition to the *LuxS* gene, the frequencies of the six virulence genes in the 87 strains of *A. veronii* isolates were as follows: 2.30% (2/87); 21.14% (21/87); 25.29% (22/87); 26.44% (23/87); 14.94% (5/87); 5.75% (5/87); and 1.15% (1/87) of the isolates contained 6, 5, 4, 3, 2, 1, and 0 types of virulence genes, respectively. The number of strains carrying 0 to 4 virulence genes as isolated in summer outnumbered those isolated carrying 0 to 4 virulence genes when in spring. However, there were a higher number of strains that carried 5 to 6 virulence genes when isolated in spring rather than in summer (Table 5). Strains carrying 0 to 1 virulence genes isolated in the supermarkets outnumbered those isolated in aquatic markets. However, a higher number of strains carrying 2–6 virulence genes was isolated in aquatic markets than in supermarkets. Overall, strains isolated in aquatic markets carried a higher number of virulence genes (Table 6) than those isolated in supermarkets. It can be seen from the table that the number of virulence genes carried by *A. veronii* isolates had no correlation with sampling sites, sampling time, or fish species (Table S2).

Several virulence genes were isolated from *A. veronii* in *Carassius auratus*, *Ctenopharyngodon idella*, and *Cyprinus carpio* from both supermarkets and aquatic markets, in both spring and summer. However, no virulence genes of *A. veronii* were isolated from *Silurus asotus* in the supermarkets. No correlation existed between the virulence genes carried by *A. veronii* isolates and sampling location, sampling time, nor species of fish. Among the isolated strains, 88.51% (77/87) carried a 431 bp fragment of the *aer* gene; 71.26% (62/87) carried a 232 bp of the *act* gene; 40.23% (35/87) carried a 1082 bp fragment of the *Aha* gene; 54.02% (47/87) carried a 128 bp fragment of the *ser* gene; 51.72% (45/87) carried a 323 bp fragment of the *exu* gene; and 45.98% (40/87) carried a 247 bp fragment of the *lip* gene. *LuxS* (a marker gene for the synthesis of quorum sensing signal molecule AI-2) was present among 59.77% (52/87) of the isolated strains (Supplementary Figure S1).

### 3.4. Pathogenicity Test

One strain each of the *Aeromonas* strains carrying either 0, 1, 2, 3, 4, 5, or 6 virulence genes was randomly selected for a pathogenicity test to determine the half-lethal dose (LD50) in mice. Of all the strains, CC7282-3 showed no lethality against mice at the tested concentrations. The LD50 of SL7231-1 was 4.51 × 10^9^ CFU (Colony-Forming Units)/mL, the LD50 of CC7281-2 was 3.22 × 10^8^ CFU/mL, and the LD50 of SL7232-1 was 4.27 × 10^7^ CFU/mL. For SJ7231, the LD50 of SJ7231-3 was 1.21 × 10^7^ CFU/mL, the LD50 of SN7252-4 was 6.42 × 10^6^ CFU/mL, and the LD50 of SC4122-5 was 4.17 × 10^6^ CFU/mL (Table 7).

## 4. Discussion

*A. veronii* is widely found in the environment, especially in freshwater and estuaries, and demonstrates strong adaptability. Recent reports of infection in diverse aquatic species have prompted investigation into the bacterial pathogenesis in humans, animals, and fish [24,25]. Previously, research has shown that isolates could infect fish in the laboratory [26]. We investigated the burden of *A. veronii* infection in commercially available fish, including *Carassius auratus*, *Cyprinus carpio*, *Ctenopharyngodon idella*, and *Silurus asotus*. *Carassius auratus* and *Silurus asotus* showed strong resistance when infected in vitro with pathogenic *A. veronii* isolated from *Cyprinus carpio*. Although we successfully infected *Carassius auratus* and *Silurus asotus*, they eventually recovered without death, suggesting that the pathogenic strains of *A. veronii* can also exist in vivo in certain fish. In addition, our experimental results also confirmed that *A. veronii* existed in all of the four above-mentioned commercial fish (total separation rate of 17.84%). At the same time, according to the percentage of *A. veronii* isolated from these four freshwater fish, it can be seen that the separation rate is not related to the size of fish. Since the four types of freshwater fish are common edible fish in northern China, this investigation into the burden of *A. veronii* infection is of great public health significance. 

The detection of the 16S rDNA gene is a powerful tool for the detection and identification of pathogenic bacteria [27]. The 16S rDNA-specific PCR confirmed the in vivo presence of *A. veronii* in *Carassius auratus* and other species, while other researchers also isolated this bacterial strain from American *Silurus asotus* [28]. The isolation rate of the pathogen in different fish indicated that the infectious burden of *Carassius auratus* was the highest, which may be associated with the polyphagic and benthic characteristics of *Carassius auratus*. *Cyprinus carpio* showed strong anteversion, suggesting the ability to carry a variety of pathogenic bacteria without manifesting disease. The infectious burden of *Silurus asotus* was the lowest, which was inconsistent with the separation rate of *A. veronii* in the study of Nawaz, which was probably due to the different sample collection locations and different types of studied *Silurus asotus* species [29,30,31]. The separation rate of *A. veronii* in the summer samples showed a specific increase compared with samples collected during spring, which increased the risk of product spoilage; even fresh fish are susceptible to bacterial contamination. The consumption of these aquatic products without appropriate disinfection leads to food poisoning, warranting the monitoring of *A. veronii* infection in aquatic products during summer. In terms of sampling location, the *A. veronii* burden in samples collected in the supermarkets was significantly lower than in those collected in the aquatic markets, which was closely related to the adverse environment of fish cultivation, contaminated water, crowded conditions, and fish polyculture. In comparison, the conditions for the preservation of aquatic products in the supermarkets were better, which was due to the good water quality and aeration. Therefore, it is suggested that regulatory authorities should intensify the supervision of aquatic products that are commercially available.

The pathogenesis of *A. veronii* infection is complex and is currently under active investigation. However, the relationship between the virulence genes and the pathogenicity of bacterial strains is yet to be elucidated [32]. The detection of bacterial virulence genes is essential to determine their potential pathogenicity and prevent possible infectious disease. This study analyzed the carrying status of virulence genes in *A. veronii* strains isolated from fish intestines to verify their potential pathogenicity. The results showed that 50% of the bacterial strains isolated from fish intestine carried four or more types of virulence genes, suggesting that the aquatic products of commercially available freshwater fish in Changchun may carry pathogenic *A. veronii*, which was similar to the results of Rahman [24]. This study also confirmed that *A. veronii* isolated from the intestinal tract of fish was different from that isolated from the aquatic environment. Aerolysin is the main virulent factor in *Aeromonas*, contributing to bacterial pathogenesis [33,34]. However, in the 1990s, the aerolysin gene had not been detected in any *A. veronii* isolates [35]. In 2002, Gonzolez-Serrano also confirmed that *A. veronii* strains isolated from aqueous environment lacked the aerolysin gene [36]. In 2007, Nawaz found that the *aer* gene existed in 96% of the bacterial strains isolated from *Silurus asotus* [21]. The results of this study also show that 88.51% of *A. veronii* isolated from fish intestines carried the *aer* gene. Whether this contributes to enhanced virulence in the environment still remains to be investigated in additional strains. In addition, serine proteinase affects the activity of extracellular enzymes such as aerolysin, strongly influencing the virulence of bacterial strains. Adhesin and phospholipase play an important role in adhesion and integration, as well as in the pathogenesis of *Aeromonas* species [37,38,39]. This study found that the carrying rate of the *lip* gene in *Aeromonas* strains isolated in spring was obviously higher than in summer. However, whether this is a common phenomenon needs further analysis, as well as the determination of its relationship with pathogenicity. As a virulence factor, nuclease plays an important role in other pathogenic bacteria, although Nawaz did not detect the *exu* gene in the strains isolated from *Silurus asotus* [21]. However, this test confirmed that the bacterial isolates carried the *exu* gene (51.72%), although it was unclear whether this phenomenon was related to the regional differences between strains.

*LuxS* is the marker gene regulating the synthesis of quorum sensing signal molecule AI-2 [40,41]. The quorum sensing system is associated with bacterial virulence [42,43], and it is closely associated with biofilm production [44,45]. Studies have demonstrated that bacterial biofilm enhances resistance [46]. In this study, 59.77% of the bacterial isolates carried the *LuxS* gene, suggesting the potential for biofilm formation.

The pathogenicity test showed that the more virulence genes the strain carried, the smaller the LD50 and the stronger the pathogenicity. Thus, we could confirm that the number of virulence genes carried by *A. veronii* might be positively correlated with the pathogenicity of *A. veronii*. In summary, our research shows that there were many different strains of *A. veronii* in the intestinal and aquatic environment of fish, which might potentially cause great harm in terms of public health and safety.

In addition, the relationship between the virulence genes carried by the isolated strains and the samples and their sampling environment is not obvious, which suggests a wide prevalence of pathogenic *Aeromonas* strains in aquatic products. Due to the diversity of the virulence genes, consumers handling and cooking fresh aquatic products should be aware of any possible *A. veronii* contamination. Public health authorities should monitor the potential pathogenesis of *A. veronii* for effective prevention and control measures.

## 5. Conclusions

The results showed that nearly 50% of the *A. veronii* isolates carried at least four or more virulence genes, and 25% of the *A. veronii* isolates carried at least five or more virulence genes. And there was no significant relationship between the type and quantity of virulence genes carried by the isolates and the sampling environment, time and sample type. The *A. veronii* isolates carrying more virulence genes were found to be more virulent in mice. The commercial fish carrying *A. veronii* poses potential threat to human health.

## Figures and Tables

**Table 1:** Species, quantity, and origin of fish sampled in spring.

Fish Species	Supermarket						Aquatic Market					
	1	2	3	4	5	Total	1	2	3	4	5	Total
*Carassius auratus*	3	3	2	4	3	15	2	2	3	2	3	12
*Ctenopharyngodon idella*	3	4	2	3	3	15	3	3	2	2	4	14
*Cyprinus carpio*	2	2	3	4	3	14	2	2	3	2	3	12
*Silurus asotus*	2	0	2	2	0	6	2	1	2	2	1	8
Total	10	9	9	13	9	50	9	8	10	8	11	46

**Table 2:** Species, quantity, and origin of fish sampled in summer.

Fish Species	Supermarket						Aquatic Market					
	1	2	3	4	5	Total	1	2	3	4	5	Total
*Carassius auratus*	3	3	3	3	2	14	2	3	2	3	4	14
*Ctenopharyngodon idella*	3	2	3	3	3	14	3	3	3	2	3	14
*Cyprinus carpio*	4	3	3	2	2	14	3	3	2	3	4	15
*Silurus asotus*	3	2	3	2	3	13	3	2	3	2	4	14
Total	13	10	12	10	10	55	11	11	10	10	15	57

**Table 3:** Polymerase chain reaction (PCR) primers and target fragment size.

Target Gene	PCR Primer Sequence (5′–3′)	Size of Target Fragments (bp)	Annealing Temperature (°C)
16S rDNA	P1: GGGATAACTACTGGAAACGGTA P2: GAAGGCACTCCCGTATCTCTA	886	56
*aer*	P1: CCTATGGCCTGAGCGAGAAG P2: CCAGTTCCAGTCCCACCACT	431	56
*act*	P1: GAGAAGGTGACCACCAAGAACA P2: AACTGACATCGGCCTTGAACTC	232	60
*ser*	P1: CTCCTACTCCAGCGTCGGC P2: GATCGTCGGTGCGGTTGT	128	64
*Aha*	P1: GGCTATTGCTATCCCGGCTCTGTT P2: CGGTCCACTCGTCGTCCATCTTG	1082	60.4
*lip*	P1: CACCTGGT(T/G)CCGCTCAAG P2: GTACCGAACCAGTCGGAGAA	247	56
*exu*	P1: AGACATGCACAACCTCTTCC P2: GATTGGTATTGCC(C/T)TGCAA	323	56
*LuxS*	P1: GATCCTCTCCGAGGCGTGG P2: AGGCTTTTCAGCTTCTCTTCC	369	58

**Table 4:** Distribution of *A. veronii* isolates.

Fingerling	Supermarkets		Aquatic Markets		Separation RRate
	Spring	Summer	Spring	Summer	
*Carassius auratus*	3/31 (9.68%)	5/30 (16.67%)	8/33 (24.24%)	14/34 (41.18%)	23.44%
*Ctenopharyngodon idella*	3/30 (10.00%)	4/32 (12.50%)	9/29 (31.03%)	10/30 (33.33%)	21.49%
*Cyprinus carpio*	3/29 (10.34%)	5/31 (16.52%)	7/31 (22.58%)	8/31 (25.81%)	18.85%
*Silurus asotus*	0/33 (0.00%)	2/31 (6.45%)	2/30 (6.67%)	4/31 (12.90%)	6.40%

Number of *A. veronii* isolates/total isolates (separation rate %).

**Table 5:** Comparison of virulence genes of *A. veronii* isolates in different seasons.

Season	Number of Species Carrying Virulence Genes						
	0	1	2	3	4	5	6
Spring	0 (0.00%)	2 (5.71%)	4 (11.43%)	6 (17.14%)	9 (25.71%)	12 (34.29%)	2 (5.71%)
Summer	1 (1.92%)	3 (5.77%)	9 (17.31%)	17 (32.69%)	13 (25.00%)	9 (17.31%)	0 (0.00%)

**Table 6:** Comparison of virulence genes of *A. veronii* isolated from different sampling locations.

Location	Number of Species Carrying Virulence Genes						
	0	1	2	3	4	5	6
Supermarkets	1 (3.70%)	3 (11.11%)	4 (14.81%)	8 (29.63%)	6 (22.22%)	5(18.52%)	0 (0.00%)
Aquatic markets	0 (0.00%)	2 (3.33%)	9 (15.00%)	15 (25.00%)	16 (26.67%)	16 (26.67%)	2 (3.33%)

**Table 7:** Pathogenicity test results for mice in subject to *A. Veronii* infection.

Strains Name	Virulence Genotype	LD50 (CFU·mL^−1^)
CC7282-3	*aer* ^−^ *ser* ^−^ *act* ^−^ *Aha* ^−^ *exu* ^−^ *lip* ^−^	\
SL7231-1	*aer* ^+^ *ser* ^−^ *act* ^−^ *Aha* ^−^ *exu* ^−^ *lip* ^−^	4.51 × 10^9^
CC7281-2	*aer* ^+^ *ser* ^+^ *act* ^−^ *Aha* ^−^ *exu* ^−^ *lip* ^−^	3.22 × 10^8^
SL7232-1	*aer* ^+^ *ser* ^+^ *act* ^+^ *Aha* ^−^ *exu* ^−^ *lip* ^−^	4.27 × 10^7^
SJ7231-3	*aer* ^+^ *ser* ^+^ *act* ^+^ *Aha* ^+^ *exu* ^−^ *lip* ^−^	1.21 × 10^7^
SN7252-4	*aer* ^+^ *ser* ^+^ *act* ^+^ *Aha* ^+^ *exu* ^+^ *lip* ^−^	6.42 × 10^6^
SC4122-5	*aer* ^+^ *ser* ^+^ *act* ^+^ *Aha* ^+^ *exu* ^+^ *lip* ^+^	4.17 × 10^6^

## Supplementary Materials

The following are available online at https://www.mdpi.com/2076-2615/10/4/608/s1, Figure S1: The detection result of seven major virulence genes (*aer, act, Aha, ser, exu, lip, LuxS*) of the isolated strains (part). Table S1: Biochemical and physiological characteristic of strains. Table S2: Virulence genes of *A. veronii* isolated.
