# Supplementary material for: Aeromonas veronii Infection in Commercial Freshwater Fish: A Potential Threat to Public Health
Source: Animals (Basel). 2020 Apr 2;10(4):608. doi: 10.3390/ani10040608 (PMC7222775; doi:10.3390/ani10040608)
Supplement: Supplementary file 1 [file animals-10-00608-s001.pdf]

Supplementary

Table S1. Biochemical and physiological characteristic of strains.

Projects	Results	Projects	Results
Oxidase	＋	Lactose	－
H_2S	－	Maltose	＋
Dynamic experiment	＋	Mannite	＋
Gelatin liquefaction	d	Cellobiose	d
Citrate	d	Salicin	d
Inositol	－	Esculin	＋
Methyl red	＋	Sorbierite	－
V-P test	＋	Sucrose	＋
Indole	＋	Rhamnose	－
Lysine decarboxylase	＋	Arabinose	－
Ornithine decarboxylase	d	Fructose	d
0% NaCl	＋	Xylose	－
6% NaCl	－	Arginine dihydrolase	＋
Glucose	＋		

Note: The "＋" means positive reaction; the "－" means negative reaction; the "d" means differences exist between strains.

Table S2. Virulence genes of *A. veronii* isolated.

Carassius auratus——**Spring , Aquatic markets**								
**Serial number**	**Strains name**	*lip*	*exu*	*ser*	*Aha*	*aer*	*act*	*LuxS*
1	SJ4091-4	+	-	+	-	+	+	-
2	SJ4092-2	-	+	-	-	-	-	-
3	SJ4092-5	+	-	-	+	-	+	-
4	SJ4101-2	+	-	+	-	+	+	+
5	SJ4102-1	+	+	+	-	+	+	-
6	SJ4102-3	+	+	-	-	+	+	-
7	SJ4102-4	+	-	-	-	+	+	+
8	SJ4102-5	+	+	+	+	+	+	-
Total		7	4	4	2	6	7	2
Carassius auratus——**Spring , Supermarkets**								
**Serial number**	**Strains name**	*lip*	*exu*	*ser*	*Aha*	*aer*	*act*	*LuxS*
9	CJ4121-1	-	+	-	-	+	+	+
10	CJ4121-3	-	+	+	-	-	-	+
11	CJ4131-2	-	-	+	-	-	-	-

Total		0	2	2	0	1	1	2

Carassius auratus **——Summer , Aquatic markets**

**Serial number**	**Strains name**	***lip***	***exu***	***ser***	***Aha***	***aer***	***act***	***LuxS***
12	SJ7211-1	+	+	-	+	+	+	-
13	SJ7212-1	+	-	-	-	+	+	-
14	SJ7212-3	+	-	+	+	+	+	+
15	SJ7213-2	+	-	-	+	+	+	+
16	SJ7213-5	+	+	+	-	+	+	+
17	SJ7231-1	-	-	-	-	+	+	-
18	SJ7231-3	-	-	+	+	+	+	+
19	SJ7232-2	-	+	+	+	+	+	-
20	SJ7232-5	-	-	+	-	+	+	+
21	SJ7233-1	-	-	+	-	+	+	-
22	SJ7233-5	-	+	+	-	+	+	+
23	SJ7292-3	-	+	+	-	+	+	+
24	SJ7292-4	-	-	+	-	+	-	-
25	SJ7291-2	+	-	-	+	+	+	-
Total		6	5	9	6	14	13	7

Carassius auratus **——Summer , Supermarkets**

**Serial number**	**Strains name**	***lip***	***exu***	***ser***	***Aha***	***aer***	***act***	***LuxS***
26	CJ7251-3	+	-	-	+	+	-	+
27	CJ7251-4	+	+	+	-	+	+	-
28	CJ7251-5	+	+	-	+	+	+	-
29	CJ7252-1	-	-	-	-	+	-	+
30	CJ7252-2	-	-	+	-	+	+	+
Total		3	2	2	2	5	3	3

Ctenopharyngodon idella **——Spring , Aquatic markets**

**Serial number**	**Strains name**	***lip***	***exu***	***ser***	***Aha***	***aer***	***act***	***LuxS***
31	SC4121-1	+	-	-	+	-	-	-
32	SC4121-2	+	+	-	+	+	+	+
33	SC4121-4	+	-	+	+	+	-	-
34	SC4121-5	+	+	-	+	+	+	+
35	SC4122-2	+	-	+	+	+	+	+
36	SC4122-4	+	+	+	-	+	+	+
37	SC4122-5	+	+	+	+	+	+	+
38	SC4123-1	+	+	-	+	+	-	+

39	SC4123-3	+	-	+	+	+	+	+
Total		9	5	5	8	8	6	7

Ctenopharyngodon idella——**Spring , Supermarkets**

**Serial number**	**Strains name**	*lip*	*exu*	*ser*	*Aha*	*aer*	*act*	*LuxS*
40	CC4151-1	-	-		-	+	+	+
41	CC4151-3	+	-	+	-	+	+	-
42	CC4152-4	+	-	-	-	+	-	-
Total		2	0	1	0	3	2	1

Ctenopharyngodon idella——**Summer , Aquatic markets**

**Serial number**	**Strains name**	*lip*	*exu*	*ser*	*Aha*	*aer*	*act*	*LuxS*
43	SC7251-1	-	+	+	+	+	+	-
44	SC7251-5	-	-	+	-	+	+	-
45	SC7252-1	-	-	+	-	+	-	-
46	SC7252-4	-	+	+	+	+	+	+
47	SC7261-1	-	+	+	-	-	-	+
48	SC7261-3	-	+	+	-	-	-	+
49	SC7262-1	-	+	+	+	+	+	+
50	SC7271-1	-	-	+	-	+	+	-
51	SC7271-3	-	+	+	-	+	+	-
52	SC7272-5	-	-	+	+	-	-	+
Total		0	6	10	4	7	6	5

Ctenopharyngodon idella——**Summer , Supermarkets**

**Serial number**	**Strains name**	*lip*	*exu*	*ser*	*Aha*	*aer*	*act*	*LuxS*
53	CC7281-2	-	-	-	-	+	+	-
54	CC7281-5	+	-	-	+	+	-	+
55	CC7282-3	-	-	-	-	-	-	+
56	CC7282-4	-	+	-	-	-	-	+
Total		1	1	0	1	2	1	3

Cyprinus carpio——**Spring , Aquatic markets**

**Serial number**	**Strains name**	*lip*	*exu*	*ser*	*Aha*	*aer*	*act*	*LuxS*
57	SL4131-1	-	-	+	+	+	+	+
58	SL4131-3	+	+	+	-	+	+	-
59	SL4132-1	+	-	+	+	+	+	-
60	SL4132-2	-	-	-	+	+	+	+
61	SL4132-5	+	+	-	+	+	+	-

62	SL4141-2	+	-	-	-	-	+	-
63	SL4131-5	+	+	+	-	+	+	-
Total		5	3	4	4	6	7	2

***Cyprinus carpio*——Spring , Supermarkets**

**Serial number**	**Strains name**	***lip***	***exu***	***ser***	***Aha***	***aer***	***act***	***LuxS***
64	CL4251-1	+	+	+	-	+	+	+
65	CL4251-4	+	+	-	+	+	+	+
66	CL4252-2	+	+	+	-	+	+	-
Total		3	3	2	1	3	3	2

***Cyprinus carpio*——Summer , Aquatic markets**

**Serial number**	**Strains name**	***lip***	***exu***	***ser***	***Aha***	***aer***	***act***	***LuxS***
67	SL7231-1	-	-	-	-	+	-	-
68	SL7231-3	-	+	+	-	+	+	+
69	SL7231-5	-	-	-	-	+	+	+
70	SL7232-1	-	-	+	-	+	+	+
71	SL7232-4	-	-	-	+	+	+	-
72	SL7232-5	-	+	+	-	+	-	+
73	SL7233-2	-	+	-	-	+	-	-
74	SL7233-4	-	+	-	-	+	+	+
Total		0	4	3	1	8	5	5

***Cyprinus carpio*——Summer , Supermarkets**

**Serial number**	**Strains name**	***lip***	***exu***	***ser***	***Aha***	***aer***	***act***	***LuxS***
75	CL8051-1	-	-	+	+	+	+	-
76	CL8051-4	-	+	+	-	+	+	-
77	CL8052-1	-	+	+	-	+	+	+
78	CL8052-2	+	-	-	-	+	+	+
79	CL8052-5	+	+	-	-	+	+	-
Total		2	3	3	1	5	5	2

***Silurus asotus*——Spring , Aquatic markets**

**Serial number**	**Strains name**	***lip***	***exu***	***ser***	***Aha***	***aer***	***act***	***LuxS***
80	SN4251-2	-	+	-	-	+	+	-
81	SN4252-3	+	+	-	+	+	-	+
Total		1	2	0	1	2	1	1

***Silurus asotus*——Spring , Supermarkets**

Total		0						

Silurus asotus——**Summer , Aquatic markets**

**Serial number**	**Strains name**	*lip*	*exu*	*ser*	*Aha*	*aer*	*act*	*LuxS*
82	SN7251-1	-	+	+	-	-	+	-
83	SN7251-4	-	+	+	+	+	-	-
84	SN7251-5	-	+	+	+	+	-	-
85	SN7252-4	-	+	+	+	+	+	+
Total		0	4	4	3	3	2	1

Silurus asotus——**Summer , Supermarkets**

**Serial number**	**Strains name**	*lip*	*exu*	*ser*	*Aha*	*aer*	*act*	*LuxS*
86	CN8052-1	+	-	-	-	+	+	+
87	CN8052-5	-	-	-	+	+	+	+
Total		1	0	0	1	2	2	2
Total		40	45	47	35	77	62	52

Note:The "＋" means positive reaction; the "－" means negative reaction.

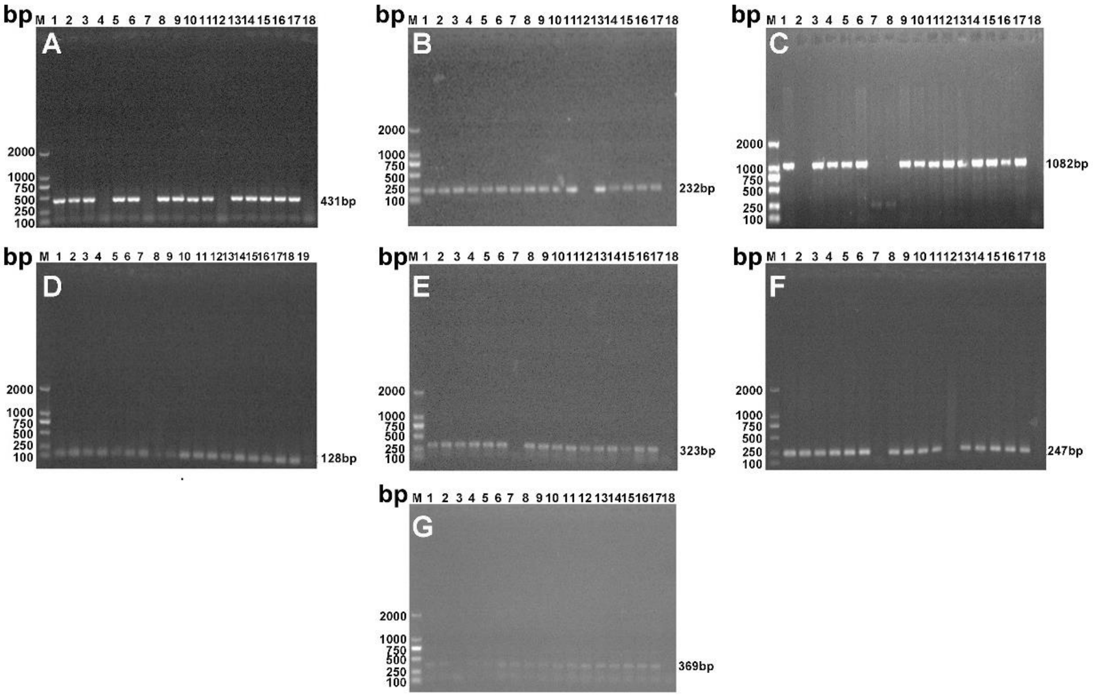

Supplementary Figure 1. The detection result of seven major virulence genes (*aer, act, Aha, ser, exu, lip, LuxS*) of the isolated strains (part).
A, *aer* detection result; B, *act* detection result; C, *Aha* detection result; D, *ser* detection result; E, *exu* detection result; F, *lip* detection result; G, *LuxS* detection result; lane 17 in A,B,C,E,F,G : positive control; lane 18 in D : positive control; lane 18 in A,B,C,E,F,G : negative control; lane 19 in D : negative control; lane M : DNA marker.
